# Fast Disinfection of *Escherichia coli* Bacteria Using Carbon Nanotubes Interaction with Microwave Radiation

**DOI:** 10.1155/2013/458943

**Published:** 2013-03-30

**Authors:** Samer M. Al-Hakami, Amjad B. Khalil, Tahar Laoui, Muataz Ali Atieh

**Affiliations:** ^1^Department of Chemical Engineering, KFUPM, Dhahran 31261, Saudi Arabia; ^2^Department of Biology, KFUPM, Dhahran 31261, Saudi Arabia; ^3^Department of Mechanical Engineering, KFUPM, Dhahran 31261, Saudi Arabia

## Abstract

Water disinfection has attracted the attention of scientists worldwide due to water scarcity. The most significant challenges are determining how to achieve proper disinfection without producing harmful byproducts obtained usually using conventional chemical disinfectants and developing new point-of-use methods for the removal and inactivation of waterborne pathogens. The removal of contaminants and reuse of the treated water would provide significant reductions in cost, time, liabilities, and labour to the industry and result in improved environmental stewardship. The present study demonstrates a new approach for the removal of *Escherichia coli* (*E. coli*) from water using as-produced and modified/functionalized carbon nanotubes (CNTs) with 1-octadecanol groups (C_18_) under the effect of microwave irradiation. Scanning/transmission electron microscopy, thermogravimetric analysis, and FTIR spectroscopy were used to characterise the morphological/structural and thermal properties of CNTs. The 1-octadecanol (C_18_) functional group was attached to the surface of CNTs via Fischer esterification. The produced CNTs were tested for their efficiency in destroying the pathogenic bacteria (*E. coli*) in water with and without the effect of microwave radiation. A low removal rate (3–5%) of (*E. coli*) bacteria was obtained when CNTs alone were used, indicating that CNTs did not cause bacterial cellular death. When combined with microwave radiation, the unmodified CNTs were able to remove up to 98% of bacteria from water, while a higher removal of bacteria (up to 100%) was achieved when CNTs-C_18_ was used under the same conditions.

## 1. Introduction 

Safe drinking water is one of mankind's most basic needs. Safe drinking water is generally defined as water that does not pose any health risk to humans. The World Health Organization (WHO) defines safe drinking water as water that has chemical, microbial, and physical characteristics that comply with both WHO guidelines for drinking water quality and the respective country's drinking water standard. Good-quality water (i.e., water free of contaminants) is essential to human health and is a critical feedstock in a variety of key industries, including the oil and gas, petrochemical, pharmaceutical, and food industries. The available supplies of water are decreasing due to (i) low precipitation, (ii) increased population growth, (iii) more stringent health-based regulations, and (iv) competing demands from a variety of users, for example, industrial, agricultural, and urban development. Consequently, water scientists and engineers are seeking alternative sources of water. These alternative sources include seawater, storm water, wastewater (e.g., treated sewage effluent), and industrial wastewater. Water recovery/recycle/reuse has proven to be effective and successful in creating a new and reliable water supply while not compromising public health [1]. Waterborne pathogens are a primary public health concern in developing countries and lead to millions of deaths per year [1]. Worldwide, waterborne diseases remain the leading cause of death in many developing nations. According to the 2004 WHO report, at least one-sixth of the world's population (1.1 billion people) lack access to safe water. The consequences are daunting: diarrhoea kills approximately 2.2 million people every year, mostly children under the age of 5 [2]. 

Most of the remediation technologies available today, while effective, very often are costly and time-consuming methods. *E. coli* is a bacterium of enteric origin whose occurrence and abundance allow for its use in defining the sanitary quality of water and wastewater. WHO has established a maximum level of 1000 faecal coliform units (FCU)/100 mL for Category A water quality [3]. Chlorination is the most widely used wastewater disinfection method, even though it has drawbacks due to the formation of trihalomethanes and organochlorinated compounds, which are carcinogens. An alternative disinfection method is the use of some metals, either alone or combined, such as Fe, Cu, or Ag in the solid state [4, 5], in ionic form [6–11], in combination with UV light [12], or as formulations in which metal ions of Al, Cu, or Ag are added to a solid matrix, such as zeolites [13, 14], ceramic material [15], silicates [16], colloids, and metal nanoparticles [17–19]. These metal particles cannot be feasibly used directly in water treatment because their toxicity to humans is not yet known, although their elemental forms are toxic to humans at high levels of exposure. However, our experiences using metals for disinfecting wastewater are limited, and they mainly involve using metal ions in combination with other chemical disinfectants, such as chlorine, hydrogen peroxide, or peracetic acid (PAA). These combinations of disinfectants have been applied to influents from advanced primary treatment (APT), biological effluents, or raw water [20–23]. Advances in nanoscale science and engineering suggest that many of the current problems involving water quality could be resolved or greatly ameliorated using nanosorbents, nanocatalysts, bioactive nanoparticles, nanostructured catalytic membranes, and nanoparticle-enhanced filtration, among other products and processes resulting from the development of nanotechnology [24]. Over the last twenty years, carbon nanotubes (CNTs) have received considerable attention from many researchers due to their interesting properties and wide range of applications. In addition to their outstanding mechanical characteristics, CNTs exhibit excellent electrical and thermal properties. These superior properties provide exciting opportunities to produce advanced materials for new applications [25–35]. Because nanoparticles (NPs), CNTs in particular, have the ability to slip past the immune system or directly into the brain or blood cells, some highly qualified research centres around the world are looking into connecting machines to individual cells to provide treatment, inject drugs, and perform many tasks related to health issues [36]. The effect of CNTs on bacteria and viruses has not received much attention, most likely due to the difficulty of dispersing CNTs in water. The antimicrobial activity of CNTs requires direct contact between CNTs and the target microorganisms [37]. Because CNTs are highly hydrophobic materials, this finding suggests that the suspension of nonfunctionalised CNTs in water is very difficult and does not provide enough CNT-microbe contact for disinfection. Few studies available have credited SWNTs with antimicrobial activity towards Gram-positive and Gram-negative bacteria, and the damages inflicted were attributed to either a physical interaction or oxidative stress that compromises the cell membrane integrity. CNTs may therefore be useful for inhibiting microbial attachment and biofouling formation on surfaces [38–41]. For example, Kang [37] immobilised SWNTs on a membrane filter surface and observed 87% *E. coli* die-off in 2 hours. Srivastava et al. [42] showed that CNTs could be incorporated into hollow fibres and achieve effective inactivation of *E. coli* and poliovirus. Brady-Estévez and Flimelech [43] achieved complete retention and effective inactivation of *E. coli* and up to 5–7 log removal of MS2 bacteriophages using a PVDF microporous membrane coated with a thin layer of SWNTs. In most cases, to achieve total inactivation of test microorganisms, the contact time tends to be large, that is, up to 2 h [36]. 

The interaction of microwaves with CNTs is an interesting topic for a variety of potential applications. Microwaves with CNTs have been used for the inactivation of microorganisms, providing a technique for simple, green, and large-scale water purification. As an innovative application, the combination of microwaves with well-aligned CNTs produced a new technology for water treatment. Moreover, the microwave-absorbing properties of CNTs and their different behaviour from typical organic compounds may open the door to the preparation of a wide range of new materials useful in many fields. The present study provides a new approach for the removal of *E. coli* from water using modified and nonmodified CNTs with and without the heating effect of microwave radiation. 

## 2. Experimental Methods and Materials

### 2.1. Carboxylation Treatment of Carbon Nanotubes

**Figure 1:**
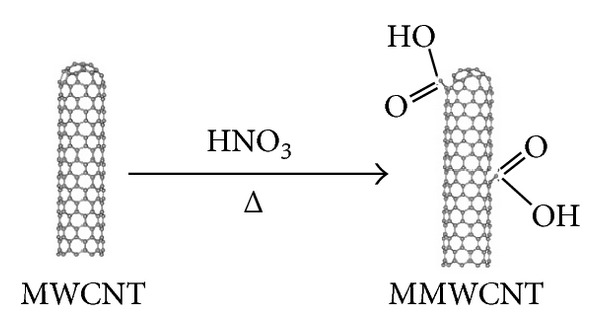
Chemical modification of carbon nanotubes through thermal oxidation [44].

Multiwall carbon nanotubes (CNTs) were purchased from Nanostructured & Amorphous Materials, Inc., USA. The purity of CNTs is >95% and the outside and inside diameters are 10–20 nm and 5–10 nm, respectively. The length of these CNTs is 10–30 *μ*m. Three hundred millilitres of a concentrated nitric acid of AnalaR (69%) was added to 2 g of as-received CNTs. The mixture was refluxed for 48 h at 120°C and then cooled to room temperature. The mixture was diluted with 500 mL of deionised water and then vacuum-filtered through a filter paper (3 *μ*m porosity). This washing operation was repeated until the pH became the same as the deionised water pH and was followed by drying in a vacuum oven at 100°C. Such conditions lead to the removal of the catalysts from the CNTs, opening of the tube caps, and the formation of holes in the sidewalls, followed by an oxidative etching along the walls with the concomitant release of carbon dioxide. This less vigorous condition minimised the shortening of the tubes, and the chemical modification was then limited mostly to the opening of the tube caps and the formation of functional groups at defect sites along the sidewalls. The final products were nanotube fragments whose ends and sidewalls were decorated with various oxygen-containing groups (mainly carboxyl groups) (Figure 1) [44]. Moreover, the percentage of carboxylic functions on the oxidised CNT surface does not exceed 4%, which corresponds to the percentage of CNT structural defects [15, 16].

### 2.2. Esterification of Carbon Nanotubes

The Fischer esterification is an equilibrium reaction, whereas other esterification routes do not involve equilibrium. To shift the equilibrium to favour the production of esters, it is customary to use an excess of one of the reactants, either the alcohol or the acid. In the present reactions, an excess of 1-octadecanol (Merck, 97% purity) was used because it is cheaper and easier to remove than the CNTs. Additionally, water formed in this reaction was removed by evaporation during the reaction.

**Figure 2:**
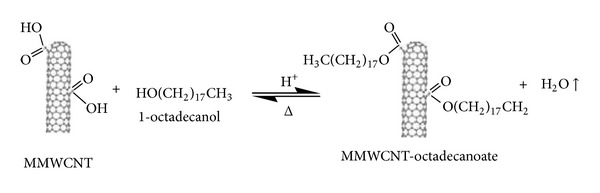
Chemical esterification of modified carbon nanotubes (M-CNTs) [44].

 The oxidatively introduced carboxyl groups represent useful sites for further modifications [17], as they enable the covalent coupling of molecules through the creation of ester (Figure 2) [44]. In a 250 mL beaker, 10 g of the 1-octadecanol was melted on a hotplate at 90°C, and 1 g of oxidatively modified carbon nanotubes (M-CNTs) was added. The mixture was stirred for 10 minutes before a few drops of sulphuric acid were added as a catalyst. After the addition of catalyst, the reaction was kept on a hotplate and stirred for 2 hours. After completion of the reaction, the mixture was poured into 250 mL of benzene and vacuum-filtered through a filter paper (3 *μ*m porosity). This washing operation was repeated five times and was followed by washing with petroleum ether three times and THF three times. The product was washed with deionised water and acetone a few times, and then the functionalised M-CNT material that was produced was dried in a vacuum oven at 90°C.

The mechanism for this reaction involves the nucleophilic addition of the alcohol or amine to the carbonyl group of the protonated acid in carbon nanotubes, followed by elimination of a proton. The tetrahedral intermediate is unstable under the acidic conditions of the reaction and undergoes dehydration to form the ester or amide. The key steps of this mechanism involve activation of the carbonyl group by protonation of the carbonyl oxygen, nucleophilic addition to the protonated carbonyl to form a tetrahedral intermediate, and elimination of water from the tetrahedral intermediate to restore the carbonyl group.

### 2.3. FTIR Measurements

Fourier transform infrared (FTIR) spectroscopy has shown a limited ability to probe the structure of CNTs. A factor that has hindered the advancement of FTIR spectroscopy as a tool for CNT analysis is the poor infrared transmittance of CNTs. To overcome this problem, KBr preparations of nanotube samples were utilized. Because of their black character, the CNTs exhibit strong absorbance and are often unable to be distinguished from background noise, for that a very low concentration of CNTs in a KBr powder was used. However, the greater vibrational freedom of attached polymeric species produces much more pronounced peaks, and so the attached species are typically the focus of attention in FTIR results. Despite this, with very careful sample preparation, some researchers have managed to elucidate peaks corresponding to surface-bound moieties, such as carboxylic acid groups at wavenumbers of 1791, 1203, and 1080 cm^−1^. The spectra of samples were recorded by a PERKIN ELMER 16F PC FTIR instrument. FTIR samples were prepared by grinding ~0.03 wt% dry material into potassium bromide. 

### 2.4. Thermal Analysis

The thermogravimetric analysis (TGA) technique measures the changes in the weight of a sample with increasing temperature. The moisture content and presence of volatile species can be determined with this technique. Computer-controlled graphics can calculate the weight percent losses. The dynamic thermogravimetric experiments were performed using a Netzsch model STA 449 F3 Jupiter simultaneous thermal analyser, which allows measurement of the mass change and the associated phase transformation energetics. The system was equipped with a PtRh furnace capable of operation from 25 to 1500°C. The temperature was measured with a type S thermocouple. The system is vacuum tight, allowing measurements to be conducted under a controlled atmosphere. Differential scanning calorimetry (DSC) measurements were also taken to study the phase transitions and exothermic/endothermic decompositions taking place in the investigated samples. The TGA-DSC analysis was performed on small samples (approximately 6 mg) mounted on platinum crucibles with Al_2_O_3_ liners and pierced lids in a flow of inert atmosphere (flow rate of argon gas, 70 mL/min) for the degradation study and under air for the same conditions (flow rate of air gas, 70 mL/min) for the oxidation study. The temperature range was varied from room temperature to 1400°C, and the typical heating rate was 20°C/min, while the digital resolution of the balance is 1 *μ*g/digit. 

### 2.5. Surface Characterisation of CNTs

The morphological and structural study of CNTs was conducted using field-emission scanning electron microscopy (JEOL JSM-6700F) and transmission electron microscopy (Philips CM200-FEG). To prepare TEM samples, some alcohol was dropped on the nanotube film, which was transferred with a pair of tweezers to a carbon-coated copper grid.

### 2.6. Microorganisms and Culture Conditions

The *E. coli* strain ATCC number 8739 (supplied by King Fahd University, Petroleum and Minerals Clinic) was used throughout this work. *E. coli* was grown overnight in nutrient broth at 37°C on a rotary shaker (160 rpm). Aliquots of the pre-culture were inoculated into fresh medium and incubated in the same conditions to an absorbance at 600 nm of 0.50. The cells were harvested by centrifugation at 4000 rpm for 10 min at 4°C, washed twice with a sterile 0.9% NaCl solution at 4°C, and dispersed in the solution containing CNTs and CNTs-C_18_ to a concentration of 3.5 × 10^7^ CFU/mL.

The bactericidal rate *K* can be calculated by the following formula:

(1) $$\begin{array}{c} K=\frac{\left(A-B\right)}{A}\times 100\%, \end{array}$$

where *K* is The bactericidal rate; *A* is colony forming units (CFU)/mL of the control sample; *B*  is colony forming units (CFU)/mL of the tested sample. The optimum bactericidal rate can be accounted in each Petri dish should be in the range of that  30–300 CFU/mL.

### 2.7. Microwave Application

**Figure 3:**
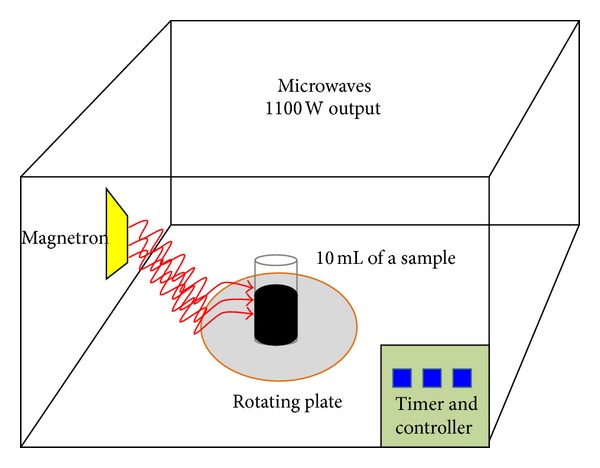
Illustration of experimental setup: the microwaves with 1100W output power was provided with a rotating plate, so that the radiation circumference covered an entire sample under study.

The above solution of nanomaterial was sonicated before being mixed with the bacterial solution. For each type of carbon nanomaterial used, the mixture of carbon nanomaterial and bacteria was tested with and without exposure to microwave radiation for 0, 5, and 10 seconds (Figure 3). Cultured bacteria (tested bacteria with different carbon nanomaterials with and without microwave radiation) were analysed by plating on nutrient agar plates after serial dilution in 0.9% saline. The colonies were counted after a 48 h incubation at 37°C. *E. coli* control experiments were performed in parallel with each CNTs-C_18_ material tested.

## 3. Results and Discussion

The present study reports the results obtained using CNTs and CNTs functionalised with 1-octadecanol groups (C_18_) for the removal of *E. coli* bacteria dispersed in water as well as the additional effect of using microwave radiation. 

### 3.1. Characterisation of Carbon Nanotubes

**Figure 4:**
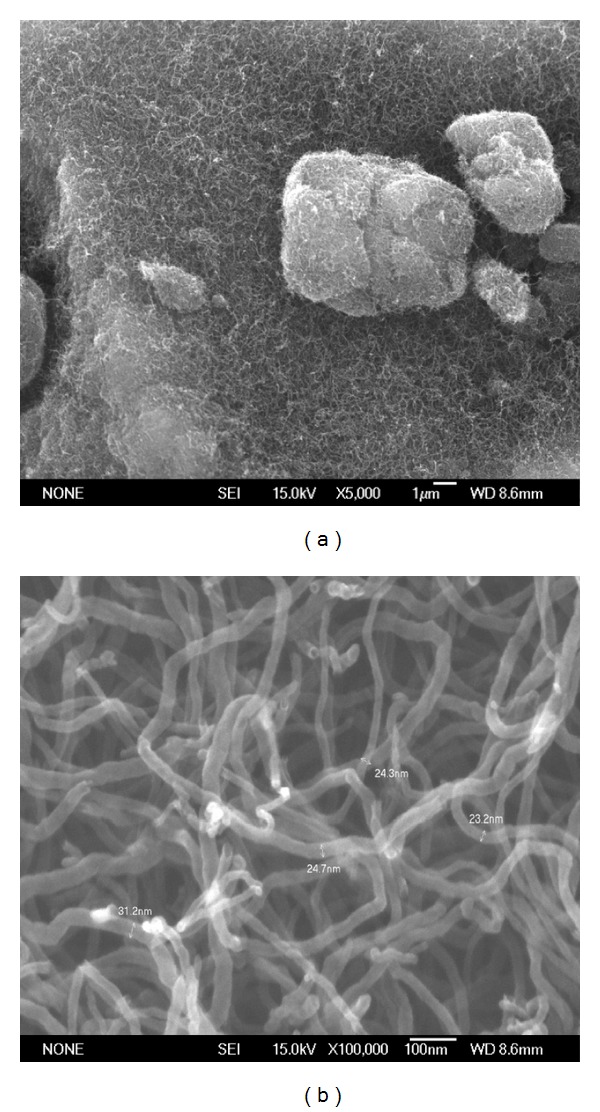
SEM Images of carbon nanotubes at (a) low resolution (b) high resolution.

**Figure 5:**
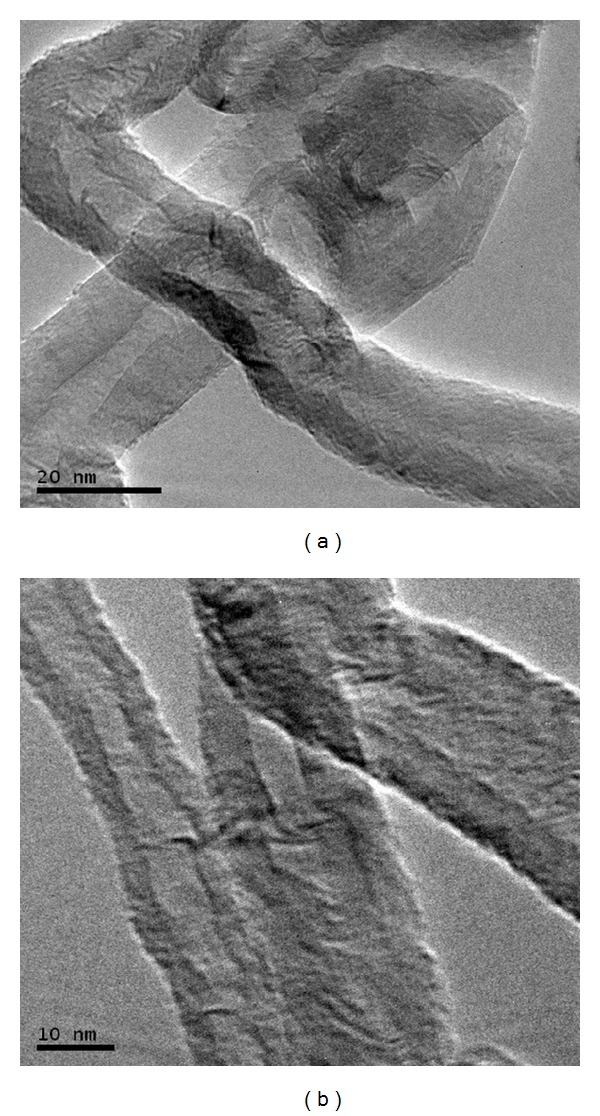
TEM images of CNTs at (a) low resolution and (b) high resolution.

The CNTs were observed by FE-SEM and TEM. The diameter of the CNTs was found to vary from 20 to 40 nm with an average diameter at 24 nm, while the length of the CNTs reached up to a few microns.  Figure 4(a) shows SEM image of CNTs at low magnification, while Figure 4(b) shows a higher-magnification image. From the SEM observation, the product represents relatively high-quality CNTs. TEM was also performed to characterise the structure of the nanotubes (Figure 5). To prepare TEM samples, some alcohol was dropped onto the nanotube film, which was transferred with a pair of tweezers to a carbon-coated copper grid. It is obvious from the images that all the nanotubes are hollow and tubular in shape. In some of the images, catalyst particles can be observed inside the nanotubes.  Figure 5(b) shows the high-resolution transmission electron microscopy (HRTEM) of the CNTs and that a highly ordered crystalline CNT structure is present. 

### 3.2. Surface Modification of Carbon Nanotubes

**Figure 6:**
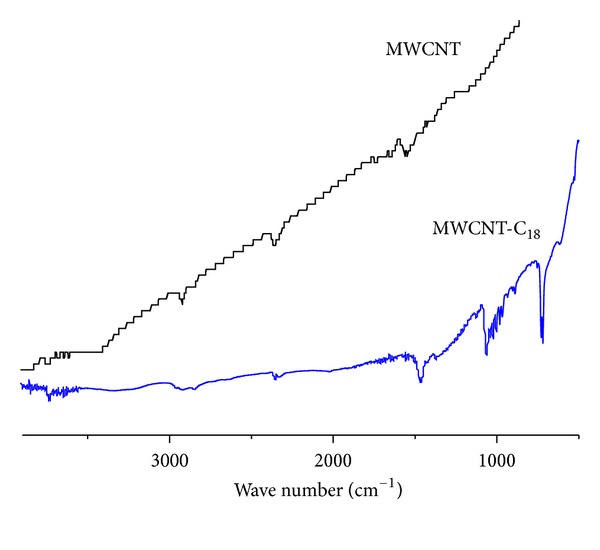
FTIR spectra of MWCNT and 1-octadecanol (C_18_) modified MWCNT.

Figure 6 shows the IR spectra of as-received multiwall CNTs and CNTs functionalised with 1-octadecanol (CNT-C_18_). The IR spectrum of MWCNTs shows an absorption band at 2920 cm^−1^, which is attributed to asymmetric and symmetric CH_2_ stretching. The band 1698 cm^−1^ is assigned to carboxylic  C=O stretching, and 1097 cm^−1^ corresponds to C–O stretching in alcohols. The presence of these functional groups on the surface of pure MWCNTs indicates their introduction during the removal of metal catalysts in nanotube purification processes. The carboxylic C=O stretching peak observed at 1693 cm^−1^ can be attributed to an acid treatment of MWCNTs, which is reported elsewhere. Substitution of the MWCNT-COOH group with 1-octadecanol gives an indicative peak at 2920 cm^−1^ corresponding to CH_2_ stretching of the long alkyl molecule of octadecanol. The peaks 1473, 1458, and 1068 cm^−1^ correspond to the ether formation of octadecanol with the –COOH groups in CNTs.

### 3.3. Thermal Oxidation and Degradation of Modified and Unmodified CNTs

**Figure 7:**
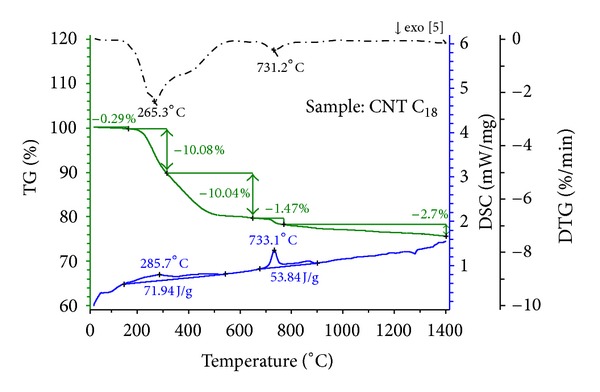
Temperature-dependent mass change (TG), rate of mass change (DTG), and heat flow rate (DSC) of the sample CNT-C_18_.

**Figure 8:**
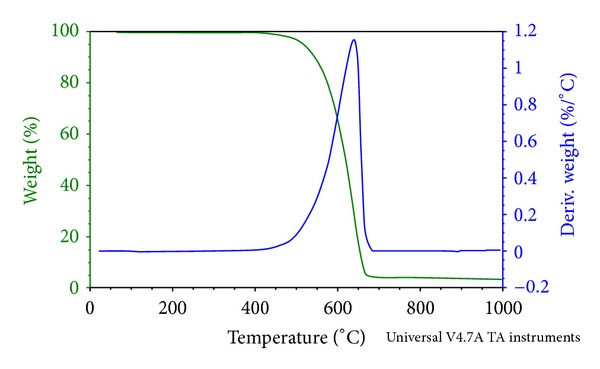
Temperature-dependent mass change (TG), rate of mass change (DTG) of raw CNTs.

The study of the thermal degradation of materials is of major importance because it can, in many cases, determine the upper temperature limit of use for a material. In addition, considerable attention has been directed towards the exploitation of thermogravimetric data for the determination of functional groups. For this purpose, thermogravimetric analysis (TGA) is a technique widely used because of its simplicity and the information afforded by a simple thermogram.  Figure 7 depicts the TGA-DSC results for the CNTs functionalised with amine groups (CNT-C_18_). In these figures, TG% refers to the temperature-dependent mass change in percent, DTG (%/min) to the rate of mass change (derivative of TG curve), and DSC (mW/mg) to the heat flow rate of the considered sample. Several mass loss steps were observed, which are due to the release of moisture (below ~150°C) and the decomposition of the associated organic groups. The mass loss steps were accompanied by endothermic effects visible in the DSC signal except for the sample.  Figure 7 displays the degradation of CNT-C_18_. Two peaks appear at 265°C and approximately 400°C, corresponding, respectively, to the maximum degradation of C_18_ and the carboxylic acid group. From Figure 8, it seems that a small amount of phenol has been attached to the carboxylic group, therefore yielding a small DTG peak appearing at approximately 250°C corresponding to the maximum degradation of phenol followed by the peak at 376°C showing the maximum degradation of the carboxylic group. It is well known that carbon will decompose in atmospheric environment. For that, the samples were burned under air atmosphere to reveal the purity of raw carbon nanotubes. The TG thermograms were carried out in air, and it was noted that there was some residual remains of the samples when they was heated to approximately 900°C, as shown in Figure 8, this residual being the catalyst. It can be observed that this decomposition process is a single-stage decomposition reaction in which the procedural decomposition temperatures are well defined.

### 3.4. Effect of CNTs and Functionalised CNTs on the Removal of *E. coli* Bacteria

This novel nanomaterial consisting of CNTs functionalised with 1-octadecanol (C_18_H_38_O) or simply C_18_ group was investigated thoroughly in this study. The dosing amount of the CNTs-C_18_ during all experiments was fixed at 0.2 g of CNTs/100 mL of NaCl autoclaved solution. Table 1 presents the percentage of *E. coli* bacteria removed by adding CNTS and CNTs-C_18 _nanomaterial. 

As shown in Table 1, the results indicate that the percentage of *E. coli* bacteria removal by CNTs and CNTs-C_18_ is relatively small (3–5%), which could be due to experimental error, meaning no significant removal occurred. The antimicrobial activity of CNTs requires direct contact between CNTs and target microorganisms [41]. Our experiments provide direct evidence that no major removal of *E. coli* bacteria would take place if the as-received CNTs or CNTs modified by carboxylic or 1-octadecanol (C_18_H_38_O) functional groups were used. 

### 3.5. Removal of *E. coli* Bacteria by Microwave Radiation Interaction with CNTs

**Figure 9:**
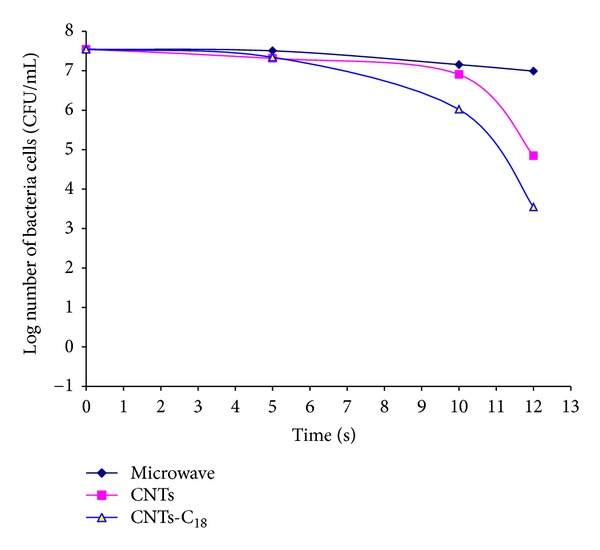
Effect of CNTs and functionalised CNTs-C_18_ with and without microwave radiation on removal of bacteria from drinking water.

In this investigation, microwaves were utilised as a source of heat for the removal of *E. coli* bacteria. The disinfection of *E. coli* bacteria from drinking water before and after treating it with CNTs and functionalised CNTs with and without microwave radiation is shown in Figure 9. Under microwave radiation alone, the die-off was very limited, approximately 0.04 to 0.4 log reduction of the number of bacterial cells after 5 and 10 sec, respectively, while using microwaves in the presence of CNTs gave a reduction of approximately 0.2 to 0.6 log. A higher reduction was obtained with CNTs-C_18_, yielding approximately 0.2 to 1.5 log after 5 and 10 sec, respectively. Increasing the radiation time to 12 sec, a sharp reduction in the number of bacterial cells was obtained using CNTs-C_18_, yielding a reduction of 4 log (99.99% removal), 2.7 log (98.9%) with CNTs, and 0.6 log (72%) with microwave radiation alone. The bacterial die-off, which was enhanced after using microwave heating, might be due to the effect of polarisation on the cell wall. This polarisation is expected to exert acute and excessive potentials and heat, leading to bursting of the cell wall. 

**Figure 10:**
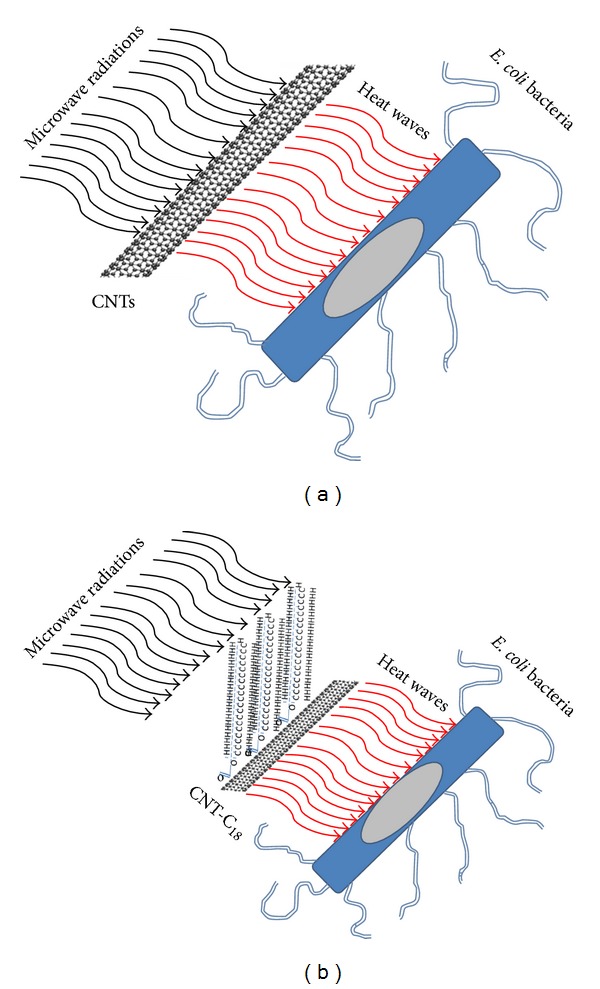
Schematic diagram of the thermal effect CNTs and CNT-C_18_ under microwave radiation on *E.coli*.

Figure 10 shows the schematic diagram of the removal of the *E. coli* bacteria under the effect of microwave radiation using as-received and functionalised CNTs. The functionalisation of CNTs with C_18_ (C–C bonds) groups provides further enhancement of the thermal properties of CNTs due to the long carbon chains, which increased the absorption rate of the microwave heat attached to the surface of CNTs, leading to higher heat absorption and thus an overall increased temperature, particularly after a prolonged time period (12 sec). 

## 4. Conclusion

The interaction of microwaves with carbon nanotubes (CNTs) is in fact an interesting topic and has a wide range of recent applications. The present study highlights a new approach for the removal of *E. coli* from water using as-received CNTs and CNTs modified with 1-octadecanol groups (C_18_) under the effect of microwave irradiation. The morphology of the as-received CNTs was characterised using field-emission scanning electron microscopy (FE-SEM) and transmission electron microscopy (TEM) to determine the diameter and the length of the CNTs. The diameter of the CNTs varied from 20 to 40 nm with an average diameter of 24 nm; the length was 10 micrometres. The surface of the CNTs was functionalised with 1-octadecanol (C_18_) functional groups via the Fischer esterification, which was confirmed by fourier transform Infrared (FTIR) spectroscopy, thermogravimetric analysis (TGA), and differential scanning calorimetric (DSC). The unmodified and modified CNTs were tested for their efficiency in destroying the pathogenic bacteria from water, with and without the effect of microwave radiation. A low removal (3–5%) of *E. coli* bacteria was observed when CNTs alone were used, indicating that the CNTs alone do not cause bacterial death. A high removal of *E. coli* bacteria was obtained when microwave radiation was used. Almost complete removal of *E. coli* bacteria (100%) was obtained using CNTsC_18_ followed by CNTs (98. %) and then a microwave source (71%) for 12 sec. CNTs functionalised with carbon-18 functional groups with microwave radiation generally showed the highest antibacterial activity when compared with non-functionalised carbon nanotubes interacting with microwave radiation and microwave radiation without a carbon source. These significant results were obtained due to multiple chains of C_18_ (C–C bonds), which increased the absorption rate of the microwave heat. As an innovative application, the combination of microwaves with modified and unmodified CNTs appears to be promising and can complement the currently employed disinfection methods. Moreover, the microwave absorbing properties of CNTs and their unique behaviour compared with typical organic compounds may open the door to the preparation of a wide range of new materials useful in many fields. Therefore, extensive and deeper scientific research studies are needed in the field of “Bionanotechnology”, not only for human purposes but also for the environment around us (flora and fauna). Unknown illnesses, new viruses, and thousands of dangerous bacteria are filling the world, and these crucial issues are pushing scientists to deploy new techniques for illness treatment, water purification applications, and many other environmental issues (ir 23, 8670–8673).

## Figures and Tables

**Table 1:** The percentage of *E. coli* removal under the effect of CNTs and CNTs-C_18_.

Type of carbon nanotubes	Number of *E. coli* cells	% of *E. coli* removal (*K*)
	Control sample	
Raw CNTs	3.50*E* + 07	5
CNTs-C_18_	3.50*E* + 07	3
